# Recurrence Patterns after Radiotherapy for Glioblastoma with [(11)C]methionine Positron Emission Tomography-Guided Irradiation for Target Volume Optimization

**DOI:** 10.3390/diagnostics14090964

**Published:** 2024-05-05

**Authors:** Zsanett Debreczeni-Máté, Imre Törő, Mihaly Simon, Kristof Gál, Marton Barabás, David Sipos, Arpad Kovács

**Affiliations:** 1 Doctoral School of Health Sciences, Faculty of Health Sciences, University of Pécs, 7621 Pécs, Hungary; mzsani1994@gmail.com (Z.D.-M.);; 2 Department of Oncoradiology, Faculty of Medicine, University of Debrecen, 4032 Debrecen, Hungary; 3 Department of Medical Imaging, Faculty of Health Sciences, University of Pécs, 7621 Pécs, Hungary

**Keywords:** 11C MET PET, glioblastoma, recurrence pattern

## Abstract

^11^C methionine (^11^C-MET) is increasingly being used in addition to contrast-enhanced MRI to plan for radiotherapy of patients with glioblastomas. This study aimed to assess the recurrence pattern quantitatively. Glioblastoma patients undergoing 11C-MET PET examination before primary radiotherapy from 2018 to 2023 were included in the analysis. A clinical target volume was manually created and fused with MRI-based gross tumor volumes and MET PET-based biological target volume. The recurrence was noted as an area of contrast enhancement on the first MRI scan, which showed progression. The recurrent tumor was identified on the radiological MR images in terms of recurrent tumor volume, and recurrences were classified as central, in-field, marginal, or ex-field tumors. We then compared the MET-PET-defined biological target volume with the MRI-defined recurrent tumor volume regarding spatial overlap (the Dice coefficient) and the Hausdorff distance. Most recurrences occurred locally within the primary tumor area (64.8%). The mean Hausdorff distance was 39.4 mm (SD 32.25), and the mean Dice coefficient was 0.30 (SD 0.22). In patients with glioblastoma, the analysis of the recurrence pattern has been mainly based on FET-PET. Our study confirms that the recurrence pattern after gross tumor volume-based treatment contoured by MET-PET is consistent with the FET-PET-based treatment described in the literature.

## 1. Introduction

Glioblastoma multiforme (GBM) represents a formidable challenge in oncology. The characteristic histopathological features are necrosis and endothelial proliferation, resulting in grade IV malignancy, the highest in the World Health Organization (WHO) classification. It stands as the predominant primary malignant brain tumor in adults and remains among the most formidable adversaries in the realm of human cancers. Despite the implementation of aggressive multidisciplinary interventions, GBM continues to manifest dismal prognostic outcomes. Typically, patients encounter a median progression-free survival (PFS) of merely 6–7 months, coupled with an overall survival (OS) spanning 14–16 months from the point of initial diagnosis [1,2,3].

The prevailing therapeutic paradigm for GBM, as delineated by the Stupp protocol, revolves around a comprehensive approach. This strategy encompasses maximal safe surgical resection pursued by adjuvant radiotherapy (RT) complemented by concurrent and maintenance temozolomide (TMZ) chemotherapy [4,5]. However, achieving durable local tumor control poses a formidable challenge, owing to the pervasive infiltrative nature of GBM, its genetic heterogeneity, and the formidable obstacle presented by the blood–brain barrier.

The first-line treatment strategy for malignant gliomas is surgical resection. Because of the invasive nature of high-grade gliomas, total surgical removal of the tumor is almost impossible, so further treatment is always needed. The most important point is reaching correct tumor delineation based on imaging, aiming to understand the actual degree of normal tissue infiltration, and optimizing the extent of surgical resection. The therapeutic goal of surgery is to remove as much tumor tissue as safely feasible without compromising neurological function.

The first choice of diagnostic imaging modality is magnetic resonance imaging (MRI). MR imaging reveals morphological information on the size and localization of tumors and delineates secondary phenomena such as mass effect, necrosis, hemorrhage, and signs of increased intracranial pressure. Brain MRI, including T2-weighted, T2-weighted fluid-attenuated inversion recovery (FLAIR) sequences, and 3D T1-weighted sequences before and after application of a gadolinium-based contrast agent, is the diagnostic gold standard to detect a brain tumor.

External fractionated radiotherapy stands as a cornerstone in the treatment arsenal against glioblastoma, significantly enhancing overall survival [6]. The goal of radiotherapy is to improve local control without inducing neurotoxicity. However, despite this advancement, tumor recurrence invariably ensues, typically within the initial 6 months post-treatment. Recurrences predominantly manifest within the high-dose region following radiotherapy, with occurrences distanced from this zone becoming more prevalent in the later stages of the disease progression [7,8]. In the absence of a standardized therapeutic approach for recurrent GBM, the evolution of technology has paved the way for the adoption of stereotactic radiosurgery (SRS), stereotactic radiotherapy (SRT), or helical tomotherapy (HT) as a viable strategy for re-irradiation in patients with brain tumors. Several investigations have highlighted a potential survival advantage following re-irradiation in recurrent GBM patients. Nevertheless, uncertainties persist regarding the efficacy and potential toxicities associated with a secondary course of radiotherapy [9].

In the modern radiotherapy of brain malignancies, the irradiated treatment volumes’ delineation has been traditionally based on computed tomography (CT) and MRI information. The area of the resection cavity and the residual tumor area identified on T1-weighted, T2-weighted, and FLAIR MRI sequences is defined as the macroscopic target volume. In addition to the conventional imaging modalities employed for cerebral tumors, considerable emphasis is placed on the integration of positron emission tomography (PET) imaging, which provides insight into the biological and functional aspects of tumor morphology. Amino acid PET imaging, in particular, has emerged as a valuable tool for diagnostic and grading purposes, as well as for guiding surgical and radiotherapy planning and distinguishing between tumor recurrences. Among the array of tracers utilized for amino acid PET imaging, l-[Methyl-11C]-Methionine (MET) [10,11,12,13,14,15], 6-fluoro-(18F)-l-3,4-dihydroxyphenylalanine (FDOPA), and [18F] fluoroethyl-tyrosine (FET) stand out as the most extensively employed.

The primary advantage of amino acid and amino acid analog positron emission tomography (PET) tracers lies in their ability to exhibit relatively high uptake within tumor tissue while demonstrating comparatively low uptake in normal brain tissue. This property enables the precise delineation of active tumor volumes, even in regions devoid of contrast enhancement on conventional magnetic resonance imaging [13]. Leveraging multimodal imaging with 11C-MET PET alongside MRI holds promise in facilitating a more accurate identification of active tumors. Consequently, this approach allows for the delivery of higher doses to regions harboring active tumor cells, thereby necessitating smaller clinical target volume (CTV) margins. Such precision has the potential to enhance local control and overall survival outcomes among glioblastoma patients. Thanks to these* results, many centers have begun to integrate amino acid imaging into CT and MRI-based treatment planning, especially when planning highly targeted radiotherapy, in dose-escalation studies or in the treatment of recurrent disease.

In this study, the recurrence pattern of tumors in glioblastoma patients who underwent 11C-MET PET imaging was evaluated. The assessment involved a comparative analysis of the recurrent tumor volume (rGTV) with both the CTV and the biological target volume (BTV) derived from radiotherapy planning.

## 2. Materials and Methods

### 2.1. Patients Characteristics

Between January 2018 and December 2023, the University of Debrecen Clinical Centre conducted 130 MET PET scans for radiation planning purposes, out of which 44 patients were histologically confirmed to have glioblastomas. Tumor diagnoses were established through histological analysis of tissue samples obtained during biopsy or surgical resection, adhering to the diagnostic criteria outlined by the World Health Organization. The analysis specifically focused on patients who underwent 11C-MET PET examinations before commencing radiochemotherapy, with MRI and 11C-MET PET imaging available at the time of recurrence. A total of 17 patients met these criteria and were included in the analysis. Table 1 provides an overview of the patient demographics and tumor characteristics.

### 2.2. Surgery

The patients underwent surgery to remove the tumor, if this was possible. The volume of the tumor was determined by contrast-enhanced magnetic resonance imaging (post-contrast T1-weighted images, FLAIR, functional magnetic resonance imaging, and DTI). The extent of the resection was a biopsy in 2 cases, a partial resection in 12 cases, and a complete resection in 3 cases.

### 2.3. Radiotherapy

All patients underwent gadolinium contrast-enhanced MRI, encompassing T1-, T2-, and FLAIR-weighted sequences, utilizing a 3.0 Tesla Philips Achieva (Philips Healthcare, Best, the Netherlands) scanner, as part of the radiotherapy planning process. Subsequent to MRI, 11C-MET PET imaging was conducted utilizing a 64-channel Philips Gemini TF PET/CT (Philips Medical Systems) system, adhering to the radiation positioning protocol, with each patient fitted with an individualized immobilization plastic mask. CT simulation scans (3 mm in thickness at 3 mm intervals) were taken from the vertex to the inferior margin of the second cervical vertebra.

For treatment planning, CT images were co-registered with MET PET images and postoperative gadolinium-enhanced T1 sequences from the MRI. This co-registration facilitated image fusion and ensured accurate delineation of target volumes and organs at risk (OARs). Radiotherapy treatment planning was executed utilizing version 9.8 and 16.2 of the Pinnacle treatment planning system (Philips Radiation Oncology Systems, Fitchburg, WI, USA).

Radiotherapy was administered as radiochemotherapy with temozolomide, adhering to dosage parameters ranging from 2 to 60 Gy, in accordance with the protocol delineated by the EORTC 26981/NCIC CE.3 trial. The volumetric modulated arc therapy (VMAT) technique was employed to deliver radiotherapy, allowing for the optimization of radiation dose distribution while minimizing the exposure to normal brain tissue.

### 2.4. Follow-Up

Follow-up was performed by a neurosurgeon and/or clinical oncologist.

All patients attended a so-called six-week follow-up after the final report from the radiotherapy part. And 2.5–3 months after the end of radiotherapy, a contrast-enhanced cranial MR scan was performed (contrast-enhanced CT in the absence of MR), sooner in cases of symptomatic progression. In patients with macroscopic residual disease after surgery, clinical response was assessed according to response evaluation criteria in solid tumors (RECIST). In cases where pseudoprogression was suspected, an 11C-PET/CT scan was performed. If the disease progressed in the brain, patients were considered for salvage treatment (reoperation, second-line chemotherapy, or re-irradiation) on a case-by-case basis.

### 2.5. Definition of the Target Volumes

The gross tumor volume (GTV) was meticulously delineated, encompassing the resection cavity and any residual tumor visualized on postoperative contrast-enhanced T1-weighted MRI scans. GTV contouring incorporated MET PET imaging data, wherein the biological target volume (BTV), indicative of metabolically active viable tumor tissue, was demarcated. Consequently, our GTV definition integrates both MRI-derived GTV and MET PET-derived BTV. During BTV contouring, non-neoplastic structures exhibiting physiologically high 11C-MET uptake, such as blood vessels and the skull, were meticulously excluded to ensure specificity.

The clinical target volume extended beyond the GTV, incorporating a 10–20 mm margin to encompass areas of potential microscopic tumor spread. To account for possible patient positional changes, the planning target volume (PTV) was defined with an additional margin of up to 5 mm beyond the anatomically corrected CTV.

Target volume delineation was performed manually by seasoned nuclear medicine specialists and radiation oncologists, leveraging their extensive expertise to ensure precision and accuracy in defining treatment volumes. The tumor volumes of all patients are presented in Table 2 for comprehensive analysis.

### 2.6. 11C-methionine Positron Emission Tomography Data Acquisition and Analysis

The L-[methyl-11C] methionine radiotracer was synthesized utilizing an on-site cyclotron (GE PETtrace; GE Healthcare, Chicago, IL, USA). To ensure stable baseline metabolic conditions, all patients underwent a 4 h fasting period before scanning. Subsequently, patients received injections ranging from 370 to 555 MBq of 11C-methionine on the scan day.

To minimize potential confounding effects, patients were instructed to limit physical activity, followed by a 10 min rest period post-injection. Approximately 10 min after injection, low-dose transmission CT images were acquired to facilitate brain attenuation correction for the PET emission data and provide morphological information.

Whole-brain emission data were acquired in three-dimensional mode, 15–20 min post-injection of 11C-MET, utilizing a Philips Vereos Digital PET/CT scanner (Philips Healthcare, Amsterdam, the Netherlands). This comprehensive imaging protocol ensured optimal visualization and quantification of metabolic activity within the brain tissue.

### 2.7. Recurrence Pattern Analysis

The recurrence was identified as an area of contrast enhancement observed on the initial MRI scan, indicating disease progression. Axial, contrast-enhanced T1-weighted MR sequences from 17 treated patients displaying initial progression were integrated with baseline images and contours using RayStation’s (RaySearch Medical Laboratories AB, Stockholm, Sweden) automatic image registration module. Subsequently, the recurrent tumor was manually delineated based on radiological MR images, defining the recurrence gross tumor volume.

**Figure 1:**
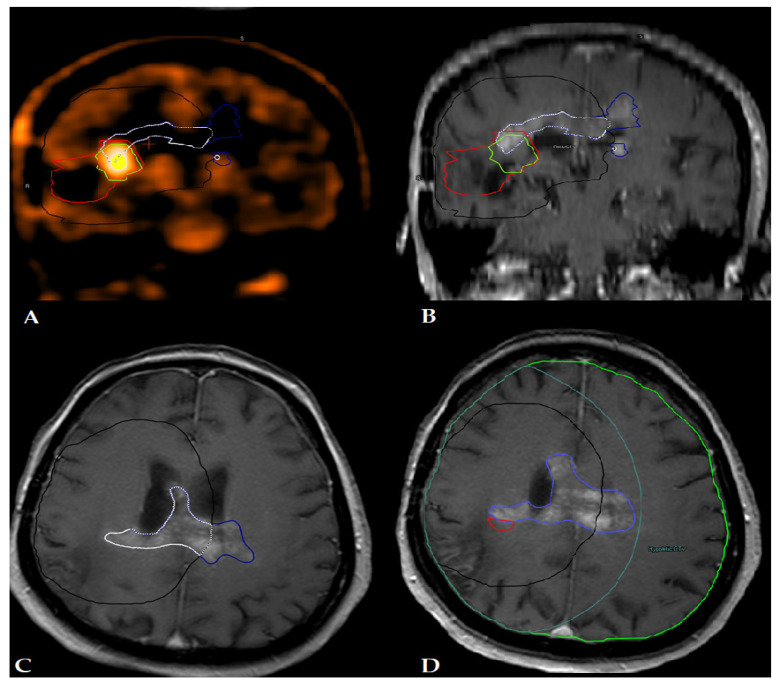
Patient number 2 shows post-contrast T1-weighted MRI (**A**,**C**,**D**) and 11C-MET PET (**B**) brain images. CTV (black), PET-MR GTV (red), and BTV (green) recurrent tumor within CTV (white), recurrent tumor outside CTV (blue), and hypothetic CTV (turquoise blue).

Radiographic response assessment was conducted in accordance with the Response Assessment in Neuro-Oncology (RANO) criteria, as outlined by Chan et al. [11]. Recurrences were categorized as central, in-field, marginal, or ex-field tumors [12]. Specifically, a recurrence was classified as central if the recurrence volume exhibited a minimum of 95% overlap with the clinical target volume. An in-field recurrence was designated as such if the recurrence volume achieved at least 80% coverage of the CTV. A marginal recurrence was identified if the recurrence volume demonstrated coverage ranging between 20 and 80% of the CTV. Conversely, an ex-field recurrence was defined as a recurrence where the volume displayed less than 20% coverage of the CTV (Figure 1).

### 2.8. Statistical Analysis

The data were statistically analyzed using 3D Slicer version 3.2.1 with the Dice Computation module used to calculate DSC and the Model to Model Distance module used to calculate HD. Excel was used for all other analyses.

Descriptive statistics were used to analyze patient characteristics. Overall survival was defined as the time from the start of radiotherapy to death. Progression-free survival was defined as the time from the start of radiotherapy to the first diagnosis of progression on MRI according to the RANO response assessment criteria.

The comparison between the biological target volume determined by MET PET scan before radiotherapy and the recurrent gross tumor volume defined by MRI at first progression was conducted using two metrics: the Dice similarity coefficient (DSC) and the Hausdorff distance (HD). The DSC ranges between 0 and 1, where 0 denotes no overlap and 1 signifies a perfect match between volumes. It quantifies the degree of overlap between the two volumes, with higher values indicating greater similarity. On the other hand, the HD measures the maximum distance between any point in one volume to the closest point in the other volume, and vice versa. Expressed in millimeters, a smaller Hausdorff distance indicates a higher degree of similarity between the volumes.

## 3. Results

At the onset of radiation therapy treatment, a cohort of 17 glioblastoma patients was enrolled, with a median age of 59 years (range of 34–77), comprising 6 males and 11 females. The median Karnofsky Performance Score (KPS) was 90, ranging from 60 to 100. Among the patients, 3 exhibited mutations in the isocitrate dehydrogenase one gene (IDH1), while 14 patients had wildtype IDH1 status. Surgical interventions varied, with 3 patients undergoing macro-total resection, 12 undergoing sub-total resection, and 2 undergoing biopsy only.

The median overall survival for the cohort was 23 months, spanning a range from 3 to 40 months, while the median progression-free survival was 8.5 months, ranging from 3 to 31 months. The essential demographic data of the patient cohort are summarized in Table 1.

The recurrence volumes demonstrated varying degrees of overlap with the clinical target volume across the patient cohort. Specifically, 11 cases exhibited a substantial overlap of at least 95% with the CTV, categorizing them as central recurrences. Conversely, none of the cases achieved a coverage of at least 80% of the CTV, precluding classification as in-field recurrences. Marginal recurrences, constituting three cases, displayed coverage ranging between 20% and 80% of the CTV, while three cases were identified as ex-field recurrences, with the renewal volume demonstrating less than 20% coverage of the CTV. There were volumetric differences between the CTV and rGTV volumes. In two cases, there was no spatial overlap between the CTV and rGTV areas. The mean overlapping area was 32.6 cm^3^, with a minimum of 0, and the highest spatial overlap between the two regions was 94.2 cm^3^. The largest recurrence lesion size detected was 123.2 cm^3^.The tumor volumes of all patients are delineated in Table 2.

Upon closer examination of the three cases characterized by marginal recurrence, it became evident that achieving complete coverage of the entire recurrent gross tumor volume with the CTV would necessitate an impractical expansion of the target volume. Such an approach would entail irradiating a considerable volume of intact brain tissue, highlighting the intricate balance required between treatment efficacy and minimizing radiation-induced toxicity to surrounding healthy brain tissue.

Our results show that the recurrence classification of Chan et al. [12] does not discriminate the distance of rGTVs from the CTV in the coverage range of 20–80%, i.e., for marginal recurrences, although beyond a certain distance, recurrences are clearly outside the radial field. In one case, the ex-field designation was significant because it allowed for additional radiation treatment outside the previous high-dose field. In this patient, the volume of the recurrence received a maximum dose of 4.3 Gy from the previous radiation treatment in 30 fractions, which corresponds to 2.3 Gy in EQD2 (2 Alpha/Beta), so we performed an additional radiation treatment with a dose of 60 Gy.

To gauge the degree of similarity between the biological target volume and the recurrent gross tumor volume, two metrics were employed: the Dice coefficient and the Hausdorff distance [13]. For the Dice coefficient, quantifying the degree of spatial overlap between the volumes, the highest value was 0.73, while the minimum value was 0, yielding a mean value of 0.30 (SD of 0.22). Meanwhile, the Hausdorff distance, reflecting the maximum distance between any voxel in one volume to the closest voxel in the other volume, resulted in a mean value of 39.4 mm, with a standard deviation of 32.25 mm (min: 16.8 mm; max: 126.7 mm). The HD95% distance between the BTV and rGTV areas was a minimum of 7.8 and a maximum of 108,6 (mean HD95% = 27.4).

## 4. Discussion

High-grade gliomas (HGGs), including glioblastomas, represent a challenging group of brain tumors due to their aggressive nature and infiltrative growth. Medical imaging techniques such as PET-CT and MRI have revolutionized assessing and managing high-grade gliomas. Integrating PET-CT’s metabolic information with MRI’s anatomical and functional data offers a comprehensive understanding of tumor biology and its relationship with surrounding structures. The value of FDG PET for central nervous system tumors in radiotherapy planning is severely limited, as the high glucose metabolism in the brain reduces the accuracy of tumor delineation. In contrast to FDG, radiolabeled amino acids such as [11C-methyl]-l-methionine (MET) [14,15,16], O-(2-[18F]-fluoroethyl)-l-tyrosine (FET) [17,18], and 3,4-dihydroxy -6-[ 18F]-Fluoro-l-phenylalanine (FDOPA) [19,20] show low uptake in normal brain tissue, allowing for the visualization of brain tumors with a high tumor background signal. Several studies have provided evidence that amino acids detect the solid mass of gliomas and metabolically active tumors more reliably than conventional imaging modalities [21,22,23,24]. The overexpression of amino acid transporters in aggressive gliomas leads to increased uptake of radiolabeled amino acids like 11C-MET, reflecting the heightened metabolic demands of these tumors. This uptake pattern enables the accurate delineation of tumor extent, including regions of infiltration not easily detectable by other imaging modalities. 11C-MET PET imaging provides crucial information for tumor grading, treatment planning, and monitoring response to therapy. It is important to note that combining MR and PET imaging modalities in the GTV does not lead to higher treatment volumes, as these can even be reduced by reducing the uncertainty of the active tumor extent [23]. This approach has the potential to achieve similar therapeutic efficacy with fewer side effects and may offer options for intensification of concomitant systemic treatment with a focus on the problem of distant failure.

11C-methionine PET imaging has been used for many years in diagnosis and staging, for planning surgery and radiotherapy, and for differentiating recurrence.

Grosu et al. reported that the dimensions and positioning of remaining MET uptake show notable distinctions from irregularities identified in postoperative MRI scans among patients who have undergone surgery for brain gliomas. Since postoperative alterations cannot be discerned from lingering tumor tissue through MRI alone, MET-PET, possessing heightened specificity for tumor matter, offers a valuable means to delineate the gross tumor volume more accurately [24].

Regarding Matsuo et al.’s findings during the radiation planning process for individuals post-GBM surgery, substantial disparities were observed between the CTV-Gd and CTV-T2 margins compared to those of CTV—[11C] MET-PET [25].

Despite aggressive multidisciplinary therapy and all of the improvements in multimodal imaging with amino acids and amino acid analog PET and in radiation treatment techniques such as volumetric-modulated arc therapy (VMAT) and intensity-modulated radiation therapy (IMRT), the overall survival of glioblastoma patients is still limited to 14–16 months [26,27]. The unfavorable prognosis is mainly due to the high propensity for local tumor recurrence. The previous analyses of recurrence patterns have shown mostly central or in-field recurrences and a low rate of marginal and ex-field recurrences [28,29]. In the present study, the recurrence pattern showed similar results, as recurrences were predominantly central, and only a few marginal and ex-field recurrences were detected. Many studies of recurrence patterns have raised the question whether the “in-field” recurrences can be reduced by dose escalation, for example by stereotactic dose escalation or by means of a simultaneous integrated boost. A recent prospective phase I study determined the maximum tolerated dose (MTD) of adjuvant volumetric modulated arc RT with a simultaneous integrated boost (VMAT-SIB) and standard dose temozolomide (TMZ) in GBM. In this trial, the MTD was 80 Gy in 25 fractions (3.2 Gy/fraction) [30]. However, when compared to historical controls and published MRI-based dose-escalation studies, no improvement in progression-free survival or overall survival was observed but there was a trend toward increased hematological and neurological toxicity. However, certain subgroups of patients may benefit from increased RT dose, while others may be more susceptible to treatment-related toxic effects. Therefore, patients who warrant higher doses and those at higher risk of toxicity may be identified through the development of predictive models. And an assessment of the impact on quality of life should be included in future trials of high-dose RT.

The idea of using MET-PET to optimize radiation dose and possibly reduce side effects remains a valid prospect despite this disappointing result. We believe that a prospective study to test whether selective dose escalation to MET-PET-positive regions could improve treatment outcomes would be useful based on the results of our study. Increasing the dose of radiotherapy, either to the entire primary tumor or to a volume within the primary tumor known to be at high risk of local recurrence as defined by MET-PET, could improve local control in patients with glioblastomas. Focal dose escalation may be more successful than uniform dose escalation over the entire contrast-enhanced MRI volume because higher doses can be safely delivered by limiting the radiation boost volume. As the recurrences largely overlapped with the primary GTV, MET-PET-based dose escalation was not expected to result in a higher burden of intact brain tissue but would provide an opportunity to increase local tumor control. Therefore, dose escalation could be recommended in brain regions distal to the brainstem.

In our study, 64.7% of patients had a central recurrence based on MET-PET-based radiotherapy planning. Therefore, it may be prudent to cautiously reduce the extension of the CTV in the future. To validate this, a large number of prospective randomized trials are certainly needed in the future.

In patients with glioblastomas, the target volume definition and the recurrence pattern analysis have been mainly based on FET-PET. One reason is that the half-life of 11C-MET is relatively short, around 20 min. This short half-life requires synthesizing the tracer on-site in a medical facility with a cyclotron.

MET-PET has been shown in several studies to accurately delineate the active tumor volume of gliomas with the same sensitivity and specificity as FET-PET.

A study by Pessina et al. showed that MET-PET allowed for the detection of areas at higher risk of recurrence located in T2-weighted FLAIR sequence abnormalities [31].

D‘Souza et al. stated that 11C-MET PET/CT and MRI demonstrated strong diagnostic capabilities in detecting residual/recurrent tumors in high-grade gliomas following therapeutic interventions [28].

### Limitations

While the present study outlined above provides valuable insights into the utility of PET-CT and MRI in assessing and managing high-grade gliomas, several limitations should be acknowledged. This study’s sample size is relatively small, consisting of only 17 glioblastoma patients. This study was conducted at a single institution, which may restrict the diversity of patient demographics, tumor characteristics, and treatment approaches. Multicenter studies are needed to validate the findings across different populations and clinical settings. The retrospective design of this study introduces inherent limitations, such as reliance on pre-existing data and potential inconsistencies in data collection and analysis. Prospective studies with standardized protocols would offer more robust evidence. While this study emphasizes the advantages of PET-CT and MRI integration, it is essential to acknowledge the potential limitations of these imaging modalities. Variations in imaging protocols, equipment quality, and interpretation may influence the accuracy and reliability of the results. This study primarily focuses on imaging-based assessments, such as tumor volume overlap and similarity metrics. While these are important surrogate endpoints, clinical outcomes such as survival, progression-free survival, and treatment response should also be considered to provide a comprehensive evaluation of the imaging techniques’ efficacy.

## 5. Conclusions

In this retrospective study, clinical and radiotherapy planning data from glioblastoma patients who underwent 11C-MET PET imaging prior to primary radiotherapy with temozolomide were examined, which compared the recurrent tumor volume with the clinical target volume from radiotherapy planning. The optimal target volume for glioblastoma radiotherapy planning has been controversial to date. According to current standards, target volume concepts are based on postoperative MRI scans, which, however, result in relatively large target volumes. As 11C-MET PET-guided RT provides precise information on the extent of metabolically active tumors, selected areas could be treated with reduced RT margins. At the same time, marginal recurrence rates are kept to an absolute minimum. In this context, the mean dose to normal brain tissue could be reduced, which is clinically relevant as a recent study correlated higher normal brain doses with shorter PFS and OS [32]. The integration of PET-CT and MRI in high-grade glioma treatment planning offers a comprehensive and detailed assessment of tumor characteristics, aiding clinicians in making informed decisions about the most effective treatment strategies. These advanced imaging techniques play a crucial role in enhancing the accuracy of diagnosis, treatment, and monitoring, ultimately improving the quality of life for patients with high-grade gliomas.

In patients with glioblastoma, the analysis of the recurrence pattern has been mainly based on FET-PET. Our study confirms that the recurrence pattern after GTV-based treatment contoured by MET-PET is consistent with the FET-PET-based treatment described in the literature.

In the future, we plan to map the progression described by MR to MET-PET to see if there is a discrepancy between MR-based rGTV and MET-PET-based rGTV.

We also want to investigate whether there is a correlation between SUV values and relapses by looking at SUV values in the parts of the rGTV that do not overlap with the BTV, as there is a chance that we will obtain higher values where we do not manually contour the BTV, and thus relapse will occur there.

Furthermore, it would be useful to refine our current recurrence classification system with respect to the significant heterogeneity of marginal recurrences.

## Figures and Tables

**Table 1:** Patient characteristics. GBM patients (n = 17) treated with chemoradiation with temozolomide after 11C MET PET imaging.

Characteristics	Patients N (%)
Sex	
Male	6 (35.3%)
Female	11 (64.7%)
Median age in years (range)	59 (34–77)
Median KPS (range)	90 (60–100)
IDH mutational status	
IDH1 mutated	3 (17.6%)
IDH1 wildtype	14 (82.4%)
Type of surgery	
Macro-total resection	3 (17.6%)
S-ub-total resection	12 (70.6%)
Biopsy only	2 (11.8%)

**Table 2:** Tumor and target volumes are shown in cm^3^.

Patient No.	BTV	_PET-MR_ GTV	CTV	rGTV	rGTV within CTV
1	93.4	116.0	428.9	44.2	44.2
2	17.5	37.7	336.9	11.9	6.3
3	34.0	38.8	143.9	0.5	0.0
4	22.3	67.4	197.6	14.9	14.9
5	13.8	79.0	284.6	14.9	2.6
6	80.7	64.7	198.5	26.3	26.3
7	20.9	41.5	181.3	10.3	10.3
8	43.2	21.4	153.7	123.2	94.2
9	31.3	86.5	250.6	49.7	49.7
10	48.8	57.4	234.1	61.1	61.1
11	71.1	89.0	303.0	72.0	72.0
12	6.9	65.0	252.0	51.0	51.0
13	38.4	73.6	208.2	35.4	35.4
14	109.7	102.7	319.5	67.0	67.0
15	45.9	64.1	330.5	7.6	0.0
16	35.3	24.0	187.0	29.0	15.0
17	7.7	57.0	205.0	4.6	4.6

## Data Availability

The datasets generated and/or analyzed during the current study are not publicly available due to privacy, confidentiality, and other restrictions.
